# Publisher Correction: A *Sarcina* bacterium linked to lethal disease in sanctuary chimpanzees in Sierra Leone

**DOI:** 10.1038/s41467-021-22494-5

**Published:** 2021-03-26

**Authors:** Leah A. Owens, Barbara Colitti, Ismail Hirji, Andrea Pizarro, Jenny E. Jaffe, Sophie Moittié, Kimberly A. Bishop-Lilly, Luis A. Estrella, Logan J. Voegtly, Jens H. Kuhn, Garret Suen, Courtney L. Deblois, Christopher D. Dunn, Carles Juan-Sallés, Tony L. Goldberg

**Affiliations:** 1 Department of Pathobiological Sciences, School of Veterinary Medicine, University of Wisconsin-Madison, Madison, WI USA; 2 Department of Veterinary Science, University of Torino, Torino, Italy; 3 Tacugama Chimpanzee Sanctuary, Freetown, Sierra Leone; 4 Tai Chimpanzee Project, Max Planck Institute for Evolutionary Anthropology, Leipzig, Germany; 5 Epidemiology of Highly Pathogenic Microorganisms, Robert Koch Institute, Berlin, Germany; 6 School of Veterinary Medicine and Sciences, University of Nottingham Sutton Bonington Campus, Sutton Bonington, Leicestershire UK; 7 Twycross Zoo, Atherstone, UK; 8 Genomics and Bioinformatics Department, Biological Defense Research Directorate, Naval Medical Research Center, Fort Detrick, MD USA; 9 Leidos, Reston, VI USA; 10 Integrated Research Facility at Fort Detrick, Division of Clinical Research, National Institute of Allergy and Infectious Diseases, National Institutes of Health, Fort Detrick, MD USA; 11 Department of Bacteriology, University of Wisconsin-Madison, Madison, WI USA; 12 Noah’s Path, Elche, Spain

**Keywords:** Infectious-disease epidemiology, Clinical microbiology, Pathogens, Infection

Correction to: *Nature Communications* 10.1038/s41467-021-21012-x, published online 3 February 2021.

The original version of this Article contained an error in the legend of Fig. 3, which incorrectly read ‘hemorrhagic diathesis (**b**), and emphysematous typhlocolitis (**c**’. The correct version states ‘hemorrhagic diathesis (**a**), and emphysematous typhlocolitis (**b**’. This has been corrected in both the PDF and HTML versions of the Article.

