# Supplementary material for: A Sarcina bacterium linked to lethal disease in sanctuary chimpanzees in Sierra Leone
Source: Nat Commun. 2021 Feb 3;12:763. doi: 10.1038/s41467-021-21012-x (PMC7859188; doi:10.1038/s41467-021-21012-x)
Supplement: Supplementary file 1 — Supplementary Information [file 41467_2021_21012_MOESM1_ESM.pdf]

A *Sarcina* bacterium linked to lethal disease in sanctuary chimpanzees in Sierra Leone

Supplementary Information

Supplementary Figures

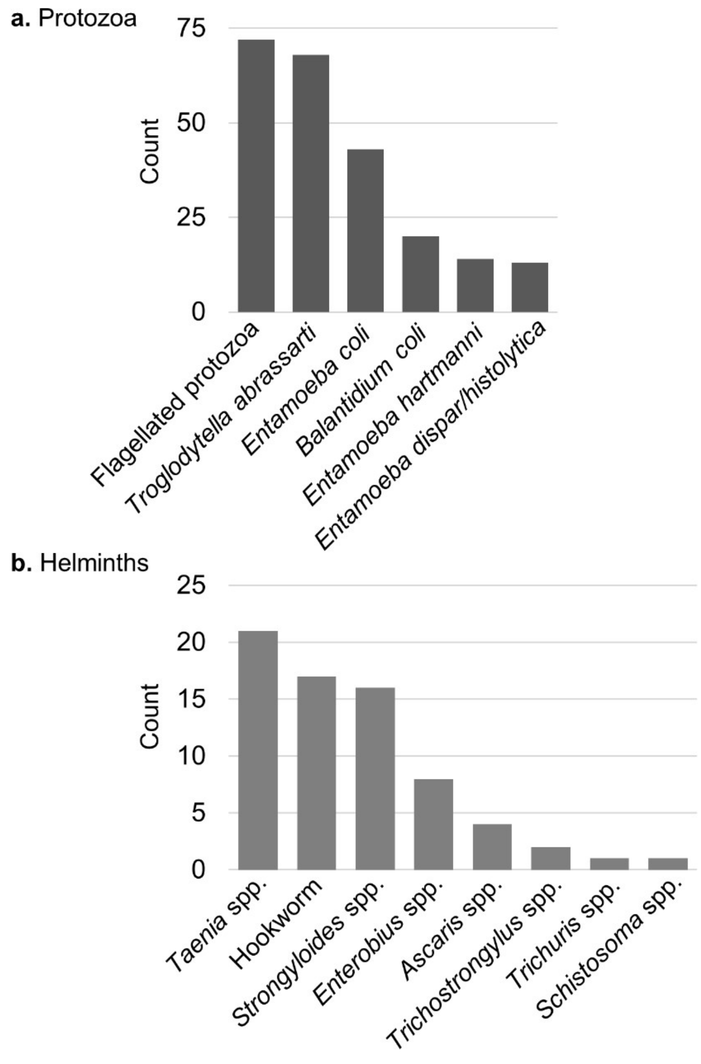

Supplementary Figure 1. Chimpanzee fecal parasitology examination results from 2005 through 2018. (a–b) Parasitology records from 17 ENGS cases and 13 controls from 2005–2018 (n = 155) are shown as a histogram in which “count” represents the number of instances a parasite was identified in an individual by any method within a 48-h period. Protozoans (a) and helminths (b) are shown separately (note different scales).

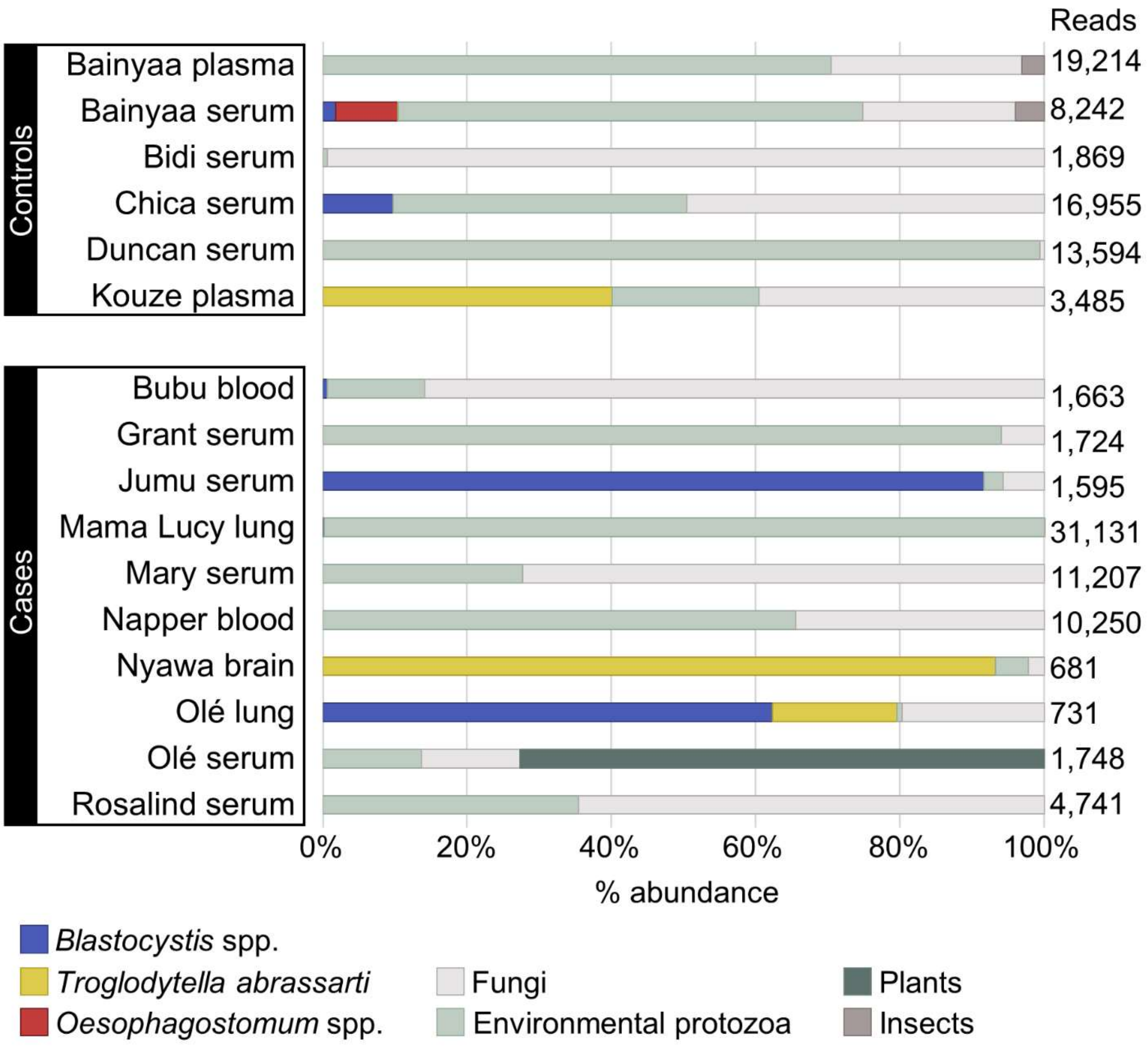

Supplementary Figure 2. Eukaryotic organisms identified with metabarcoding of the 18S rDNA gene (V9 region). Row labels indicate the name of the chimpanzee followed by the tissue type from which DNA was extracted for sequencing. The total number of reads per sample after filtering is shown on the right. Reads are grouped according to OTU and shown as percentages of total reads after filtering.

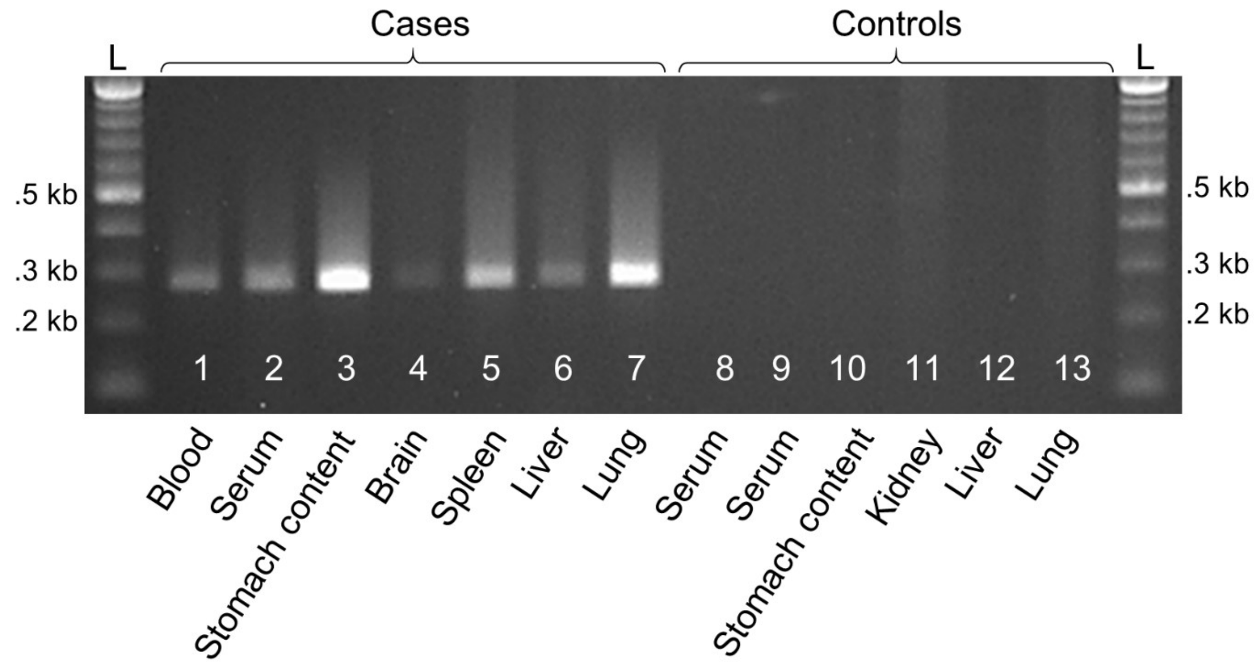

Supplementary Figure 3. **Results of diagnostic PCR for "*Ca*. S. troglodytae".** Case samples are "Nita's" post-mortem blood (1), "Mary's" serum (2), "Kafoe's" stomach content (3), "Joko's" brain (4), "Nita's" spleen (5), "Finda's" liver (6), and "Mama Lucy's" lung (7). Control samples are "Nita's" ante-mortem serum (8), "Mac's" serum (9), "Zeelie's" stomach content (10), "Gaura's" kidney (11), "Gaura's" liver (12), and "Gaura's" lung (13). 2-log DNA length standard shown in first and last lanes (L). Gel image is a representative of 3 independent experiments with similar results.

Supplementary Tables

Supplementary Table 1. Epizootic neurologic and gastroenteric syndrome (ENGS) disease characteristics.

Number of episodes of clinical signs in ENGS cases

	Number of episodes of clinical signs*				
	Min	Median	Max	Mean	n
Sudden death	0	0	0	0	24
1 episode of clinical signs preceding death	1	1	1	1	21
>1 episode of clinical signs preceding death	2	2	5	2.5	11

Duration of episodes of clinical signs in ENGS cases

	Duration of episodes in days*				
	Min	Median	Max	Mean	n
1 episode	1	6	60	10.4	21
Overall, >1 episode	1	6	90	14.2	28
Individual average, >1 episode	4.5	8.7	22.5	14.2	11

Mortality clusters within the same enclosure

	Days between cases*	
	0–2	3–4
Number of clusters	3	1

*Values were calculated using records compiled from all ENGS deaths that occurred from 2005 through 2018, with 56 cases in total.

Supplementary Table 2. 18S Metabarcoding statistics.

	All samples (n=24)					Low % samples (n=7)[c]		High % samples (n=17)[d]	
	No. raw reads[a]	Bases trimmed	% reads filtered for quality	% host reads post-quality filter	No. reads after filtering	% host reads post-quality filter	No. reads after filtering	% host reads post-quality filter	No. reads after filtering
Sum	1,477,507	210,385,414	21.45%[b]	83.32% [b]	129,443		161		129,282
Mean	61,563	8,766,059	25.25%	81.78%	5,393	89.86%	23.00	78.46%	7,604.82
Minimum	14	2,503	18.73%	10.33%	0	66.67%	0	10.33%	425
Median	66,144	9,384,788	22.24%	89.45%	1,694	92.59%	1	87.86%	3,485
Maximum	113,230	17,155,750	68.00%	100.00%	31,131	100.00%	150	96.02%	31,131
Standard deviation	31,862	4,642,950	10.95%	19.28%	7,975	10.62%	56.05	21.25%	8,579.33

[a]"Reads" refers to paired reads (all reads were paired)

[b]Overall % for combined data set

[c]Samples constituting < 0.5% total filtered reads

[d]Samples constituting > 0.5% total filtered reads

Supplementary Table 3. Viruses identified in Tacugama chimpanzees.

Virus	Accession	Genome	Closest relative (source, location, year, accession)[a]	Family[b]	Genus[b]	%ID (AA)[b]
Anellovirus	MT350347	ssDNA (circular)	Chimpanzee anellovirus (chimpanzee, Czech Republic, 2012, KT027937)	*Anelloviridae*	unclassified	90.04%
GB virus C	MT350348	ssRNA (+)	GB virus C variant troglodytes (chimpanzee, USA, 1998, AF070476)	*Flaviviridae*	*Pegivirus*	98.80%
Gemykibivirus	MT350349	ssDNA (circular)	Human associated gemykibivirus 2 (dog, Brazil, 2015, MH734235)	*Genomoviridae*	*Gemykibivirus*	99.70%
Parvovirus	MT350350	ssDNA (linear)	Parvovirus 4-like MK-2012 (chimpanzee, Cote d'Ivoire, 2002, JN798204)	*Parvoviridae*	*Protoparvovirus*	100.00%
Picobirnavirus (1)	MT350351	dsRNA (linear)	Human picobirnavirus (human, USA, 1991, AF246940)	*Picobirnaviridae*	*Picobirnavirus*	99.23%
Picobirnavirus (2)	MT350352	dsRNA (linear)	Porcine picobirnavirus (pig, India, 2013, KX374478)	*Picobirnaviridae*	unclassified	80.73%
Rhinovirus C	MT350353	ssRNA (+)	Rhinovirus C (human, USA, 2015, MG148341)	*Picornaviridae*	*Enterovirus*	98.56%
Torque teno virus (1)	MT350354	ssDNA (circular)	Torque teno virus (human, USA, 2015, KT163918)	*Anelloviridae*	*Alphatorquevirus*	59.17%
Torque teno virus (2)	MT350355	ssDNA (circular)	Torque teno virus (human, USA, 2015, KT163907)	*Anelloviridae*	unclassified	69.38%
Torque teno virus (3)	MT350356	ssDNA (circular)	Torque teno virus 14 (chimpanzee, West Africa, 2000, AB037926)	*Anelloviridae*	*Alphatorquevirus*	88.27%
Torque teno virus (4)	MT350357	ssDNA (circular)	Torque teno virus 23 (chimpanzee, Japan, 2000, NC_038342)	*Anelloviridae*	*Alphatorquevirus*	89.23%

AA, amino acid

[a]Closest match was identified by querying the viral polymerase nucleotide sequence against the NCBI's GenBank nonredundant nucleotide database using the blastn homology searching algorithm

[b]Family, genus, and percent amino acid identity refer to the closest match to the translated viral polymerase nucleotide sequence in the NCBI's GenBank nonredundant protein database

Supplementary Table 4. Culture conditions for isolation and propagation of "*Ca.* Sarcina troglodytae".

			Tissue preparation methods				
Medium	Source[a]	Catalog #	Minced with blades	Bead beat 5 min	80 °C 10 min	70 °C 10 min [1]	1:1 Ethanol [2]
AnaeroGRO BBE	HD	AG051	x[c]	x	x	na[d]	na
AnaeroGRO BRU	HD	AG301	x	x	x	na	na
AnaeroGRO CCFA	HD	AG501	x	x	x	na	na
AnaeroGRO EYA	HD	AG401	POS[e]	x	x	na	na
AnaeroGRO LKV	HD	AG601	x	x	x	na	na
AnaeroGRO PEA	HD	AG901	x	x	x	na	na
SVGM[f] pH 6.0, 1.5% agar	IH		POS	na	na	x	x
SVGM pH 6.0, 1.5% agar, 30 ml/l EYE	IH, HM	FD045	x	na	na	x	x
Willis and Hobbs Base, 30 ml/l EYE	HM	M1375, FD045	x	na	na	x	x
BHI Broth	SA	53286	x	na	na	x	x
Enriched CMB	BD	295982	x	x	x	na	na
AnaeroGRO PYEG	HD	AG24H	x	x	x	na	na
SVGM, pH 6.0	IH		x	na	na	na	na

SVGM, pH 2.2 [3]	IH		x	na	na	na	na
Thio w/H&K	HD	AG22H	x	x	x	na	na
TPGY	HM	M969	x	x	x	na	na

BBE, *Bacteroides* Bile Esculin Agar; BRU, *Brucella* Agar with Hemin and Vitamin K; CCFA, Cycloserine-Cefoxitin Fructose Agar; EYA, Egg Yolk Agar, Modified; LKV, Laked Blood with Kanamycin and Vancomycin Agar; PEA, Phenylethyl Alcohol Agar w/ 5% Sheep's Blood; SVGM, *Sarcina ventriculi* Growth Medium, EYE, Egg Yolk Emulsion; BHI, Brain Heart Infusion Broth; Enriched CMB, Cooked Meat Medium with Glucose, Hemin and Vitamin K; PYEG, Peptone Yeast Extract Glucose Broth; Thio w/H&K, Thioglycollate with Hemin and Vitamin K; TPGY, Tryptone Peptone Glucose Yeast Extract Broth

[a]Sources were Hardy Diagnostics, Santa Maria, CA, USA (HD); made in-house (IH); HiMedia, Mumbai, India (HM); Sigma Aldrich, St. Louis, MO, USA (SA); Becton Dickson, Franklin Lakes, NJ, USA (BD)

[b]Tissues attempted for culture were blood, brain, cerebrospinal fluid, colon, heart, kidney, liver, lung, muscle, pancreas, spleen, stomach content, stool, and vomit

[c]x: condition attempted, negative for "*Ca.* S. troglodytae"

[d]na: not assessed

[e]POS: condition yielded successful isolation, positive for "*Ca.* S. troglodytae"

[f]*Sarcina ventriculi* growth medium (ATCC medium 834) per liter: glucose 30 g, Bacto Peptone 5 g, yeast extract 5 g

Supplementary Table 5. Genetic distance between select pairs of bacterial species based on a 1,585-nucleotide alignment of the bacterial 16S rDNA gene.

	CP051754 “*Ca.* S. troglodytae” JB1	NR026146 *S. ventriculi*	NR026147 *S. maxima*	NR104741 *E. tarantellae*	NR112169 *C. perfringens*	NR113204 *C. perfringens*	NR121697 *C. perfringens*
“*Ca.* S. troglodytae” JB1		0.687	0.932	3.409	6.529	6.331	6.242
Sarcina ventriculi	0.216		1.363	4.049	7.187	7.054	6.868
Sarcina maxima	0.262	0.319		3.584	6.844	6.882	6.81
Eubacterium tarantellae	0.497	0.517	0.529		5.632	5.461	5.348
Clostridium perfringens	0.669	0.711	0.695	0.563		0.069	0.274
Clostridium perfringens	0.651	0.695	0.695	0.548	0.071		0.136
Clostridium perfringens	0.628	0.674	0.683	0.543	0.118	0.101	

Percent pairwise nucleotide distance is shown above the diagonal (gray), with standard error of the mean shown below the diagonal

Supplementary Table 6. Sequencing read statistics after nxtrim.

Data type[a]	No. of reads	No. of base pairs	SRA accession no.
Mate Pair	941,849	182,171,554	SRR11551921
Paired End	1,513,933	393,314,190	SRR11551922

SRA, NCBI Sequence Read Archive

[a]Mate-pair and paired end reads are shown separately, all reads originated from a single sequencing run

Supplementary Table 7. Genome statistics for "*Ca*. Sarcina troglodytae" isolate JB2.

	Accession no.	Length (base pairs)	GC%	No. ORFs[a]
Chromosome	CP051754	2,435,860	27.60%	2,223
Plasmid 1	CP051755	78,882	23.40%	98
Plasmid 2	CP051756	34,634	26.80%	42
Plasmid 3	CP051757	20,671	22.70%	20
Plasmid 4	CP051758	13,674	28.50%	17
Plasmid 5	CP051759	11,514	22.60%	11
Plasmid 6	CP051760	11,304	23.90%	10
Plasmid 7	CP051761	10,963	25.00%	14
Plasmid 8	CP051762	9,993	22.50%	10
Plasmid 9	CP051763	9,792	25.00%	12
Plasmid 10	CP051764	4,566	24.30%	4

ORF, Open reading frame

[a]Identified and annotated by PATRIC

Supplementary Table 8. "*Ca*. Sarcina troglodytae" isolate JB2 genome characteristics and comparison to the type strain *S. ventriculi* "Goodsir".

	"*Ca*. S. troglodytae" JB2	*S. ventriculi* "Goodsir"
Chromosome length (base pairs)	2,435,860	2,428,884[a]
Extrachromosomal elements	10	unknown
GC%	27.60%	27.70%
# CDS	2,223	2,252[a]
# tRNA	76	91[a]
# urease ORFs	23	0[a]
ANI to "Goodsir"	98.40%	100%[a]

ANI, average nucleotide identity

[a]This analysis is based on a manually scaffolded genome

Supplementary Table 9. Primers used in this study.

Name	Sequence (5’ – 3’)	Reference
EMP_Next_F	TCGTCGGCAGCGTCAGATGTGTATAAGAGACAGGTACACACCGCCCGTC	Belda *et al.*[4]
EMP_Next_R	GTCTCGTGGGCTCGGAGATGTGTATAAGAGACAGTGATCCTTCTGCAGGTTCACCTAC	Belda *et al.*[4]
EMP_Mammal_Block	GCCCGTCGCTACTACCGATTGGIIIIITTAGTGAGGCCCT-[C3 Spacer]	Thompson *et al.*[5]
TacuSarc_Diag_F	TGAAAGGCATCTTTTAACAATCAAAG	This study
TacuSarc_Diag_R	TACCGTCATTATCGTCCCTAAA	This study
16SV4_F	AATGATACGGCGACCACCGAGATCTACACNNNNNNNNTATGGTAATTGTGTGCCAGCMGCCGCGGTAA	Kozich *et al.*[6]
16SV4_R	GGACTACHVGGGTWTCTAATCCAGTCAGTCAGNNNNNNNNCAAGCAGAAGACGGCATACGAGAT	Kozich *et al.*[6]
16SV4_Sequencing_F	TATGGTAATTGTGTGCCAGCMGCCGCGGTAA	Kozich *et al.*[6]
16SV4_Sequencing_R	GGACTACHVGGGTWTCTAATCCAGTCAGTCAG	Kozich *et al.*[6]

NNNNNNNN, indexed barcode sequence for sample identification; I, deoxyInosine modification; -[C3 Spacer], 3-carbon phosphoramidite spacer

Supplementary References

1 Crowther, J. S. *Sarcina ventriculi* in human faeces. *J Med Microbiol* **4**, 343-350, doi:10.1099/00222615-4-3-343 (1971).

2 Edwards, A. N. & McBride, S. M. Isolating and purifying *Clostridium difficile* spores. *Methods Mol Biol* **1476**, 117-128, doi:10.1007/978-1-4939-6361-4_9 (2016).

3 Belda, E. *et al.* Preferential suppression of *Anopheles gambiae* host sequences allows detection of the mosquito eukaryotic microbiome. *Sci. Rep.* **7**, 3241, doi:10.1038/s41598-017-03487-1 (2017).

4 Thompson, L. R. *et al.* A communal catalogue reveals Earth's multiscale microbial diversity. *Nature* **551**, 457-+, doi:10.1038/nature24621 (2017).

5 Kozich, J. J., Westcott, S. L., Baxter, N. T., Highlander, S. K. & Schloss, P. D. Development of a dual-index sequencing strategy and curation pipeline for analyzing amplicon sequence data on the MiSeq Illumina sequencing platform. *Appl Environ Microbiol* **79**, 5112-5120, doi:10.1128/AEM.01043-13 (2013).

6 Tolentino, L. E., Kallichanda, N., Javier, B., Yoshimori, R. & French, S. W. A case report of gastric perforation and peritonitis associated with opportunistic infection by *Sarcina ventriculi*. *Lab. Med.* **34**, 535-537, doi:Doi 10.1309/Cdff04he9fhdqpan (2003).

7 Laass, M. W. *et al.* Emphysematous gastritis caused by *Sarcina ventriculi*. *Gastrointest. Endosc.* **72**, 1101-1103, doi:10.1016/j.gie.2010.02.021 (2010).

8 Lam-Himlin, D. *et al.* *Sarcina* organisms in the gastrointestinal tract: a clinicopathologic and molecular study. *Am. J. Surg. Pathol.* **35**, 1700-1705, doi:10.1097/PAS.0b013e31822911e6 (2011).

9 Sauter, J. L. *et al.* Co-existence of *Sarcina* organisms and *Helicobacter pylori* gastritis/duodenitis in pediatric siblings. *J Clin Anat Pathol (JCAP)* **1** (2013).

10 Tuuminen, T., Suomala, P. & Vuorinen, S. *Sarcina ventriculi* in blood: the first documented report since 1872. *BMC Infect. Dis.* **13**, 169, doi:10.1186/1471-2334-13-169 (2013).

11 Ratuapli, S. K., Lam-Himlin, D. M. & Heigh, R. I. *Sarcina ventriculi* of the stomach: a case report. *World J. Gastroenterol.* **19**, 2282-2285, doi:10.3748/wjg.v19.i14.2282 (2013).

12 Louis, G. B., Singh, P. & Vaiphei, K. *Sarcina* infection. *BMJ Case Rep.* **2014**, doi:10.1136/bcr-2013-201185 (2014).

13 Kumar, M., Bhagat, P., Bal, A. & Lal, S. Co-infection of *Sarcina* and *Giardia* in a child. *Oxf Med Case Reports* **2014**, 118-119, doi:10.1093/omcr/omu046 (2014).

14 Karakus, E. & Kirsaclioglu, C. T. Coincidence of celiac disease with *Sarcina* infection. *Turk. J. Gastroenterol.* **25 Suppl 1**, 318, doi:10.5152/tjg.2014.8028 (2014).

15 DiMaio, M. A., Park, W. G. & Longacre, T. A. Gastric *Sarcina* organisms in a patient with cystic fibrosis. *Human Pathology: Case Reports* **1**, 45-48 (2014).

16 Bhagat, P., Gupta, N., Kumar, M., Radotra, B. D. & Sinha, S. K. A rare association of *Sarcina* with gastric adenocarcinoma diagnosed on fine-needle aspiration. *J. Cytol.* **32**, 50-52, doi:10.4103/0970-9371.155238 (2015).

17 Berry, A. C., Mann, S., Nakshabendi, R., Kanar, O. & Cruz, L. Gastric *Sarcina ventriculi*: incidental or pathologic? *Ann Gastroenterol* **28**, 495 (2015).

18 Carrigan, S. *et al.* Emphysematous oesophagitis associated with *Sarcina* organisms in a patient receiving anti-inflammatory therapy. *Histopathology* **67**, 270-272, doi:10.1111/his.12599 (2015).

19 Chougule, A. *et al.* Pulmonary gangrene due to *Rhizopus* spp., *Staphylococcus aureus*, *Klebsiella pneumoniae* and probable *Sarcina* organisms. *Mycopathologia* **180**, 131-136, doi:10.1007/s11046-015-9904-3 (2015).

20 Sopha, S. C., Manejwala, A. & Boutros, C. N. *Sarcina*, a new threat in the bariatric era. *Hum. Pathol.* **46**, 1405-1407, doi:10.1016/j.humpath.2015.05.021 (2015).

21 Medlicott, S. A. C. *Sarcina ventricularis* complicating a patient status post vertical banded gastroplasty, a case. *Journal of Gastroenterology and Hepatology Research* **4**, 1481-1484 (2015).

22 Al Rasheed, M. R. & Senseng, C. G. *Sarcina ventriculi* : review of the literature. *Arch Pathol Lab Med* **140**, 1441-1445, doi:10.5858/arpa.2016-0028-RS (2016).

23 Bommannan, K., Gaspar, B. L. & Sachdeva, M. U. Pathogenic *Sarcina* in urine. *BMJ Case Rep.* **2016**, doi:10.1136/bcr-2016-216991 (2016).

24 Canan, O., Ozkale, M. & Kayaseicuk, F. Duodenitis caused by *Sarcina ventriculi* in a case with Celiac disease and selective IgA deficiency. *Cukurova Medical Journal* **42**, 766-768 (2017).

25 Mironova, M., Gobara, N., Pennell, C. P., Sherwinter, D. A. & Cimic, A. *Sarcina ventriculi:* a case report of gastric perforation in 85-year-old male with history of colon cancer. *Journal of Case Reports and Images in Pathology* **3**, 20-23 (2017).

26 de Meij, T. G., van Wijk, M. P., Mookhoek, A. & Budding, A. E. Ulcerative Gastritis and esophagitis in two Children with Sarcina ventriculi Infection. *Frontiers in medicine* **4**, 145 (2017).

27 Behzadi, J., Modi, R. M., Goyal, K., Chen, W. & Pfeil, S. *Sarcina ventriculi* as an unknown culprit for esophageal stricturing. *ACG Case Rep J* **4**, e118, doi:10.14309/crj.2017.118 (2017).

28 Rajasekar, S., Onteddu, N. & Gupta, A. A rare case of emphysematous gastritis-*Sarcina ventriculi:* 1919. *Am. J. Gastroenterol.* **113**, S1091 (2018).

29 Liu, L. & Gopal, P. *Sarcina ventriculi* in a patient with slipped gastric band and gastric distention. *Clin. Gastroenterol. Hepatol.* **16**, A25-A26, doi:10.1016/j.cgh.2017.06.042 (2018).

30 Elvert, J. L., El Atrouni, W. & Schuetz, A. N. Photo Quiz: A bacterium better known by surgical pathologists than by clinical microbiologists. *J. Clin. Microbiol.* **56** (2018).

31 Aggarwal, S. *et al.* Coinfection of *Sarcina ventriculi* and *Candida* in a patient of gastric outlet obstruction: an overloaded pyloric antrum. *Diagn. Cytopathol.* **46**, 876-878, doi:10.1002/dc.24048 (2018).

32 Shetty, N. U. *et al.* First documented case of *Sarcina* in esophageal brushing cytology. *Diagn. Cytopathol.* **46**, 886-887, doi:10.1002/dc.23986 (2018).

33 Alvin, M. & Al Jalbout, N. Emphysematous gastritis secondary to *Sarcina ventriculi*. *BMJ Case Rep.* **2018**, doi:10.1136/bcr-2018-224233 (2018).

34 Singh, K. Emphysematous gastritis associated with *Sarcina ventriculi*. *Case Rep. Gastroenterol.* **13**, 207-213, doi:10.1159/000499446 (2019).

35 Gulati, R., Khalid, S., Tafoya, M. A. & McCarthy, D. Nausea and vomiting in a diabetic patient with delayed gastric emptying: do not delay diagnosis. *Dig Dis Sci* **64**, 681-684, doi:10.1007/s10620-019-05482-0 (2019).

36 Bortolotti, P. *et al. Clostridium ventriculi* bacteremia following acute colonic pseudo-obstruction: a case report. *Anaerobe* **59**, 32-34 (2019).

37 Singh, H., Weber, M. A., Low, J. & Krishnan, U. *Sarcina* in an adolescent with repaired esophageal atresia: a pathogen or a benign commensal? *J. Pediatr. Gastroenterol. Nutr.* **69**, e57, doi:10.1097/MPG.0000000000002339 (2019).

38 Propst, R. *et al. Sarcina* organisms in the upper gastrointestinal tract: a report of 3 cases with varying presentations. *Int. J. Surg. Pathol.*, 1066896919873715, doi:10.1177/1066896919873715 (2019).

39 Dey, B., Raphael, V., Banik, A. & Khonglah, Y. *Sarcina* in sputum cytology in a patient of pulmonary tuberculosis. *J. Cytol.* **36**, 219-221, doi:10.4103/JOC.JOC_121_18 (2019).
