# Supplementary material for: A Sarcina bacterium linked to lethal disease in sanctuary chimpanzees in Sierra Leone
Source: Nat Commun. 2021 Feb 3;12:763. doi: 10.1038/s41467-021-21012-x (PMC7859188; doi:10.1038/s41467-021-21012-x)
Supplement: Supplementary file 3 — Description of Additional Supplementary Files [file 41467_2021_21012_MOESM3_ESM.pdf]

Description of Additional Supplementary Files

File Name: Supplementary Data 1
Description: **Samples obtained from chimpanzees at Tacugama Chimpanzee Sanctuary from 03/2013 to 07/2016.** These data only include frozen, unfixed samples that were available, amenable to shipping, and suitable for use in this study. Additional samples were collected during post-mortem examinations by veterinarians.

File Name: Supplementary Data 2
Description: **Comprehensive testing results.** Blank cells indicate that the test/culture was not attempted on that sample.

File Name: Supplementary Data 3
Description: **Literature review of human Sarcina cases post-1900.**
