# Supplementary material for: A Sarcina bacterium linked to lethal disease in sanctuary chimpanzees in Sierra Leone
Source: Nat Commun. 2021 Feb 3;12:763. doi: 10.1038/s41467-021-21012-x (PMC7859188; doi:10.1038/s41467-021-21012-x)
Supplement: Supplementary file 7 — Reporting Summary [file 41467_2021_21012_MOESM7_ESM.pdf]

nature research

Corresponding author(s): Tony Goldberg

Last updated by author(s): Dec 1, 2020

Reporting Summary

Nature Research wishes to improve the reproducibility of the work that we publish. This form provides structure for consistency and transparency in reporting. For further information on Nature Research policies, see our Editorial Policies and the Editorial Policy Checklist.

Statistics

For all statistical analyses, confirm that the following items are present in the figure legend, table legend, main text, or Methods section.

n/a	Confirmed	
☐	☒	The exact sample size (n) for each experimental group/condition, given as a discrete number and unit of measurement
☐	☒	A statement on whether measurements were taken from distinct samples or whether the same sample was measured repeatedly
☐	☒	The statistical test(s) used AND whether they are one- or two-sided<br>*Only common tests should be described solely by name; describe more complex techniques in the Methods section.*
☒	☐	A description of all covariates tested
☒	☐	A description of any assumptions or corrections, such as tests of normality and adjustment for multiple comparisons
☐	☒	A full description of the statistical parameters including central tendency (e.g. means) or other basic estimates (e.g. regression coefficient) AND variation (e.g. standard deviation) or associated estimates of uncertainty (e.g. confidence intervals)
☐	☒	For null hypothesis testing, the test statistic (e.g. F, t, r) with confidence intervals, effect sizes, degrees of freedom and P value noted<br>*Give P values as exact values whenever suitable.*
☒	☐	For Bayesian analysis, information on the choice of priors and Markov chain Monte Carlo settings
☒	☐	For hierarchical and complex designs, identification of the appropriate level for tests and full reporting of outcomes
☒	☐	Estimates of effect sizes (e.g. Cohen's d, Pearson's r), indicating how they were calculated

Our web collection on statistics for biologists contains articles on many of the points above.

Software and code

Policy information about availability of computer code

Data collection	Images Plus software suite v. 2.0
Data analysis	QIIME v.1.9.1, CLC Genomics Workbench v.12.0, mothur v.1.42.0, UCLUST v.0.2.0, UCHIME v.4.2, GraphPad Prism v.8.4.3, MUSCLE c.3.8.31, MEGA7 v.7.0.26, PhyML v.1.8.1, Nxtrim v.0.4.4, bbduk v.38.72, bbnorm v.3.8.9, bowtie2 v.2.3.5, SPAdes v.3.10.1, Bandage v.0.8.1, EDGE Bioinformatics v.2.3.1, PATRIC server v.3.6.2, Mauve v.2.4.0, Resistance Gene Identifier v.4.2.2, ShortBRED v.0.9.5, and the Average Nucleotide Identity Calculator (Enveomics v.0.1.3).

For manuscripts utilizing custom algorithms or software that are central to the research but not yet described in published literature, software must be made available to editors and reviewers. We strongly encourage code deposition in a community repository (e.g. GitHub). See the Nature Research guidelines for submitting code & software for further information.

Data

Policy information about availability of data

All manuscripts must include a data availability statement. This statement should provide the following information, where applicable:

- Accession codes, unique identifiers, or web links for publicly available datasets
- A list of figures that have associated raw data
- A description of any restrictions on data availability

Sequence data that support the findings of this study have been deposited in the National Center for Biotechnology Information (NCBI) GenBank database with the accession codes CP051754, CP051755, CP051756, CP051757, CP051758, CP051759, CP051760, CP051761, CP051762, CP015763, CP015764 (Bacterial genome assembly) and MT350347, MT350348, MT350349, MT350350, MT350351, MT350352, MT350353, MT350354, MT350355, MT350356, MT350357 (viral replicase genes) and in the NCBI Sequence Read Archive with the accession codes SRR11551921 – SRR115511922 (Bacterial genome sequencing reads) and BioProject accession codes PRJNA648419 (16S and 18S metabarcoding reads) and PRJNA625238 (Bacterial genome sequencing reads). Source data are provided with the

paper.

Field-specific reporting

Please select the one below that is the best fit for your research. If you are not sure, read the appropriate sections before making your selection.

- [x] Life sciences
- [ ] Behavioural & social sciences
- [ ] Ecological, evolutionary & environmental sciences

For a reference copy of the document with all sections, see nature.com/documents/nr-reporting-summary-flat.pdf

Life sciences study design

All studies must disclose on these points even when the disclosure is negative.

Sample size

Sample sizes of cases and controls were determined by the numbers and types of samples available for study. The resource-limited setting of the study site, the clinical nature of the samples, and restrictions on shipments of endangered species to the United States determined the samples that could be analyzed. All samples that could be obtained were used in this study.

Data exclusions

No data were excluded from the analyses.

Replication

The bacterium identified in this study was isolated repeatedly from frozen brain tissue plated on SVGM plates and grown in anaerobic conditions at 37 degrees Celsius. Diagnostic PCR results were confirmed at least twice (all replication attempts were successful) and all diagnostic PCR fragments were Sanger sequenced for verification. The gel image of diagnostic PCR results in Supplementary Figure 3 is a representative of 3 independent experiments with similar results. Micrographs in Figures 3c and 6a-b represent one of at least 3 replicates from the same individual with similar results. Micrographs in Figure 5a-c are from a single experiment and are representative of three independent experiments with similar results. Figure 5d shows cell size data collected from 7 distinct bacterial colonies for each bacterium.

Randomization

This was a case-control study in which randomization was not used. The study was retrospective and relied on samples which were collected opportunistically. The samples available for study were limited by the resource-limited setting in which they were collected and thus randomization was not possible.

Blinding

Investigators were blinded to sample ID for measurements of cell diameter. Samples from chimpanzee cases and controls were coded with numbers prior to processing and investigators were blind to case/control status in all experiments during data collection.

Reporting for specific materials, systems and methods

We require information from authors about some types of materials, experimental systems and methods used in many studies. Here, indicate whether each material, system or method listed is relevant to your study. If you are not sure if a list item applies to your research, read the appropriate section before selecting a response.

Materials & experimental systems

n/a	Involved in the study	
x		Antibodies
x		Eukaryotic cell lines
x		Palaeontology and archaeology
x		Animals and other organisms
x		Human research participants
x		Clinical data
x		Dual use research of concern

Methods

n/a	Involved in the study	
x		ChIP-seq
x		Flow cytometry
x		MRI-based neuroimaging
